# Describing immune factors associated with Hepatitis B surface antigen loss: A nested case-control study of a Chinese sample from Wuwei City

**DOI:** 10.3389/fimmu.2022.1025654

**Published:** 2022-10-11

**Authors:** Xiaojie Yuan, Ting Fu, Lixin Xiao, Zhen He, Zhaohua Ji, Samuel Seery, Wenhua Zhang, Yancheng Ye, Haowei Zhou, Xiangyu Kong, Shuyuan Zhang, Qi Zhou, Yulian Lin, Wenling Jia, Chunhui Liang, Haitao Tang, Fengmei Wang, Weilu Zhang, Zhongjun Shao

**Affiliations:** ^1^ Department of Epidemiology, School of Public Health, Air Force Medical University, Xi’an, China; ^2^ Ministry of Education Key Lab of Hazard Assessment and Control in Special Operational Environment, School of Public Health, Air Force Medical University, Xi’an, China; ^3^ Faculty of Health and Medicine, Division of Health Research, Lancaster University, Lancaster, United Kingdom; ^4^ Clinical Drug Experiment Institution, Gansu Wuwei Tumor Hospital, Wuwei, China; ^5^ Hepatobiliary Center, Gansu Wuwei Tumor Hospital, Wuwei, China

**Keywords:** HBsAg, interferon, chemokine, MMP-1, TNF-R1

## Abstract

**Background:**

Hepatitis B surface antigen (HBsAg) loss is considered a functional cure for chronic hepatitis B (CHB), however, several factors influence HBsAg loss.

**Methods:**

29 CHB patients who had achieved HBsAg loss, were selected and 58 CHB patients with persistent HBsAg were matched, according to gender and age (+/- 3 years). Logistic regression and restricted cubic spline (RCS) modelling were performed.

**Results:**

Multivariate-adjusted logistic regression, based on stepwise selection, showed that baseline HBsAg levels negatively correlated with HBsAg loss (odds ratio [OR] = 0.99, 95% confidence interval [CI] = 0.98-0.99). Interferon treatment positively related with HBsAg loss (OR = 7.99, 95%CI = 1.62-44.88). After adjusting for age, HBsAg level, ALT level, HBeAg status and interferon treatment, MMP-1 (OR = 0.66, 95%CI = 0.44-0.97), CXCL9 (OR = 0.96, 95%CI = 0.93-0.99) and TNF-R1 (OR = 0.97, 95%CI = 0.94-0.99) baseline levels all negatively correlated with HBsAg loss. Our multivariate-adjusted RCS model showed that baseline CXCL10 was associated with HBsAg loss although the relationship was “U-shaped”.

**Conclusions:**

Cytokines such as MMP-1, CXCL9, CXCL10 and TNF-R1 are important factors which influence HBsAg loss. It may be possible to develop a nomogram which intercalates these factors; however, further research should consider immune processes involved in HBsAg loss.

## Introduction

Approximately 316 million people worldwide suffer with chronic hepatitis B virus infections (HBV) (1) although, each country has different prevalence rates. According to a relatively recent meta-analysis, the pooled estimated prevalence of HBV infections in the Chinese mainland population, between 2013 to 2017, was thought to be 6.89%. Anything between 5.00-7.99% is officially classified as a higher intermediate prevalence (2) although again prevalence rates vary regionally. Wuwei city, which is in the north-west of mainland China, is one area in China considered to have a particularly high prevalence of chronic hepatitis B (CHB), with 7.2% (95% CI: 6.3-8.1%) in 2010 (3). As such, Wuwei city has been targeted by Chinese public health organisations as a location for pilot HBV prevention and treatment programmes.

CHB patients are at a substantially higher risk of developing cirrhosis and hepatocellular carcinoma (HCC). These diseases are responsible for approximately 1.45 million deaths worldwide, each year (4) and therefore must be prevented. Hepatitis B surface antigen (HBsAg) seroclearance can occur spontaneously in CHB patients but can also be induced with anti-viral treatments. HBsAg loss is associated with improvements in liver histologies and a decreased risk of cirrhosis, HCC development and death (5). Therefore, HBsAg seroclearance is often regarded as the optimal endpoint for treatments according to clinical guidelines (6, 7). However, HBsAg seroclearance is rare (8, 9) and therefore, we must learn to understand HBsAg seroclearance as well as the factors that influence HBsAg loss.

Previous studies have identified several factors related to HBsAg loss e.g. gender, HBV DNA levels, Hepatitis B e antigen (HBeAg) status and ALT level at baseline (10–12). Apart from the direct action of immune cells, cytokines are also thought to mediate HBsAg loss (13). At present, Interferon (IFN)-α is the main antiviral therapy for CHB and Interleukins (IL) -4, IL-6, IL-17, and IL-28 act as key “coordinators” of inflammatory responses involved in HBsAg seroclearance (13). Serum interferon-inducible protein (IP) 10, also known as C-X-C motif ligand (CXCL) 10, and CXCL13 levels could also play an important role in predicting HBsAg seroclearance (14, 15). However, the established knowledge base does not include a clear description of associations between cytokines, SNPs related to cytokines and HBsAg loss.

## Materials and methods

### Participants

From August 2018 until January 2021, 4,000 individuals, aged 30–65 years with previous HBsAg seropositive results (for at least six months), were screened in Wuwei City, China. Potential participants were asked for formal consent to participate in this study which involved a survey, a physical examination and a direct consultation with a clinician at Gansu Wuwei Tumor Hospital. After excluding duplicate data (n = 16) and those who did not complete examinations (n = 22), 3,962 participants were recruited and engaged in routine examinations every six to twelve months until 31^st^ October 2021.

All research was conducted in accordance with the Declaration of Helsinki and was approved by the Ethics Committee in the Air Force Medical University. All participants were fully informed of the purpose and details of this study, and participants (or their legal guardians) were required to provide informed consent, before participating.

The baseline for this study was defined according to the time of recruitment. The events of interest during follow-up were HBsAg loss and HBsAg seroclearance. HBsAg loss was defined as serum HBsAg which reverted to negative from positive for CHB cases with (or without) treatment. HBsAg seroclearance was defined as maintaining a HBsAg negative status on two separate occasions over six months until the endpoint. 29 participants with HBsAg loss were initially assessed using blood samples at baseline. 58 participants with persistent HBsAg(+) status were matched, according to gender and age (+/- 3 years). A flow chart of the selection and assignment process is presented in **Supplementary Figure 1.**

### Data collection

A questionnaire was specifically designed for Prevention and Control of Infectious Diseases studies in China. Trained investigators collected information through face-to-face interviews. Physical examinations were performed locally in Gansu Wuwei Tumor Hospital and results were collated. Details of examinations have been described in **Supplementary Materials, Methods**.

### Quantitative examination of HBsAg

Blood samples were obtained through standard forearm venipunctures and samples were processed within 1 h of collection. Blood samples were centrifuged at 12,000 g for 10 mins at 4°C and then supernatants were transferred into microcentrifuge tubes, which were stored at −80°C. Chemiluminescence analysis reagents (Snibe Ltd. Shenzhen, China) were used for quantitative HBsAg examinations in the Tianbo Medical Laboratory. The range was 0.02-1000 IU/mL.

### Cytokines

An RD systems Luminex^®^ Discovery Assay Human Premixed Multi-Analyte Kit (Kit Catalog Number: LXSAHM-17, Lit Lot Number: L141528) was used to detect 17 types of cytokines, including IL-6, IL-8, IL-21, IL-23, IL-33, matrix metalloproteinases (MMP)-1, MMP-2, MMP-3, C-X-C motif ligand (CXCL) 9, CXCL10, CXCL11, CXCL13, C-C motif ligand (CCL2), tumor necrosis factor (TNF) -α TNF-R1, IFN-γ, and B cell activating factor (BAFF) using a Luminex xPONENT^®^ for FLEXMAP3D^®^ analyzer. These 17 cytokines were chosen according to an associated pilot study we conducted focusing on CHB patients compared to healthy controls (in press).

A standard curve was created for each analyte through data reduction, using milliplex analyst (5.1) which is capable of generating a five-parameter logistic (5-PL) curve-fit. Cytokines below the lower limit were recorded as lower limit values. Replacement numbers for each cytokine were listed as follows: IL-8 (1/87), IL-23 (12/87), IL-33 (8/87), MMP-3 (1/87), CXCL9 (3/87), CXCL11 (6/87), TNF-α (1/87) and IFN-γ (7/87). Considering the magnitude of cytokines, we transformed MMP-1, MMP-2 and MMP-3 from pg/mL to 1000 pg/mL, and CXCL9, CXCL10, CXCL11, CXCL13, TNF-α, TNF- R1, IFN-γ, BAFF and CCL2 from pg/mL to 10 pg/mL. This enabled us to perform more sophisticated logistic regression and RCS modelling.

### Human SNPs

Genomic DNA was isolated from whole blood samples using Human Genome Whole Blood Extraction Kit (Tianlong Technology, Xi’an, China). 14 single nucleotide polymorphisms (SNP) related to cytokines (rs1061624, rs1143634, rs12979860, rs1799724, rs1799964, rs1800469, rs1800795, rs1800872, rs1800896, rs2055979, rs2069762, rs2227306, rs3025058 and rs3806798) were assessed with TaqMan^®^ MGB SNP Genotyping Kit (Fuyuan Biotechnology Ltd, Shanghai, China) using a real-time polymerase chain reaction allelic discrimination system (Tianlong Technology, Xi’an, China). Shannon entropy (SE) for human SNPs were calculated according to the following formula (16), where i means genotypes and pi means the percentage of each genotype.


$$SE=-\sum_{i}^{n}pi\times \text{In}(pi)$$

### Statistical analysis

Variables are presented as means with corresponding standard deviations (SD), or as medians with quartiles, or just as simple numbers with percentages. These data points were compared using Student’s T test, Wilcoxon’s test, or by way of a standard Chi-square tests (or Fisher’s Exact tests), according to HBsAg status. Person-time at follow-up was calculated for each participant from the date of the first survey to the end of follow-up on the 31^th^ October, 2021.

Unadjusted and multivariable-adjusted (i.e., adjusting for age, HBsAg level, ALT level, HBeAg status, and interferon treatment) logistic regression models were used to analyze associations between variables and HBsAg loss. Odds ratios (OR) with 95% confidence intervals (CI) were estimated. Restricted cubic spline (RCS) methods with three knots (p5, p50 and p95) were applied to assess the linearity of relationships using “%RCS_REG” microprogram developed by Desquilbet (17). Details of RCS modelling process are described in the **Supplementary Materials, Method Section**. RCS plots were performed with proportion distribution.

Correlations between cytokines were assessed using Spearman’s correlation analysis. To consider relations among cytokines, we established each dataset as independent and integral. Standardized cytokines were calculated according to normalized Z-scores. Partial Least Squares Discriminant Analysis (PLS-DA) and Permutational Multivariate Analysis of Variance (PERMANOVA) with Euclidean distance were also performed to compare fundamental differences between the two groups using “mixOmics” R package (http://mixomics.org/,v6.8.5) (18).

Statistical analyses were performed using SAS, version 9.4 (SAS Institute Inc, Cary, NC) and with R software, version 2.13.2 (http://cran.r-project.org/). A *p* value of 0.05 was established as the threshold for statistical significance.

## Results

### Baseline characteristics

3,962 participants were initially included at baseline, of whom 212 were HBsAg(–) and 3,750 were HBsAg(+). Among the 3,750 CHB cases with HBsAg(+), 1,694 were followed-up for a median of 1.5 years. 62 participants achieved HBsAg loss, corresponding to an annual loss rate of 2.41 per 100 person-years and with a cumulative incidence of 3.66%. Those with HBsAg loss were predominantly male, urban employees, taking antiviral treatments (specifically IFN), had a longer HBV duration since infection, and had higher levels of ALT, AST, DBIL, ALB, GGT and LSM. However, there was a small proportion of participants with HBsAg loss who were HBeAg(+) and who had lower HBV DNA loads (**Table 1**).

**Table 1:** Population characteristics for HBsAg consistence and HBsAg loss groups.

Variables	Cohort			Nested case-control		
	HBsAg consistence(n = 3,688)	HBsAg loss(n = 62)	*p*	HBsAg consistence(n = 58)	HBsAg loss(n = 29)	*p*
Age, years	46.2 ± 12.9	47.3 ± 11.2	0.487	45.8 ± 10.9	44.4 ± 11.4	0.522
Gender			0.006			1.000
Male	2160 (58.6)	47 (75.8)		42 (72.4)	21 (72.4)	
Female	1528 (41.4)	15 (24.2)		16 (27.6)	8 (27.6)	
Occupation			0.029			0.735
Agricultural worker	1771 (48.0)	27 (43.6)		25 (43.1)	10 (34.5)	
Urban employee	562 (15.2)	17 (27.4)		11 (19.0)	6 (20.7)	
Others	1355 (36.7)	18 (29.0)		22 (37.9)	13 (44.8)	
Smoker	1279 (34.7)	26 (42.0)	0.234	27 (46.6)	13 (44.8)	0.879
Drinker	570 (15.5)	13 (21.0)	0.236	13 (22.4)	4 (13.8)	0.339
Antiviral treatment			<0.001^¶^			0.307^¶^
NUC	871 (23.6)	12 (19.4)		26 (44.8)	4 (13.8)	
IFN	206 (5.6)	9 (14.5)		4 (6.9)	9 (31.0)	
Both	100 (2.7)	15 (24.2)		1 (1.7)	6 (20.7)	
None	2511 (68.1)	26 (42.0)		27 (46.6)	10 (34.5)	
IFN	306 (8.3)	24 (38.7)	<0.001	5 (8.6)	15 (51.7)	<0.001
NUC	971 (26.3)	27 (43.6)	0.265	27 (46.6)	10 (34.5)	0.283
Vaccine	821 (22.3)	17 (27.4)	0.334	13 (22.4)	13 (44.8)	0.031
Cirrhosis	220 (6.0)	4 (6.5)	0.786^¶^	8 (13.8)	2 (6.9)	0.485
Family history of HBV	1853 (50.2)	28 (45.2)	0.427	36 (62.1)	11 (37.9)	0.033
HBV duration			<0.001			0.225
≤10years	811 (22.0)	24 (38.7)		27 (46.6)	11 (37.9)	
>10 years	538 (14.6)	18 (29.0)		19 (32.8)	7 (24.1)	
Unknown	2339 (63.4)	20 (32.3)		12 (20.7)	11 (37.9)	
Anti-HBs	31 (0.8)	1 (1.6)	0.415^¶^	0 (0.0)	0 (0.0)	-
HBeAg	678 (18.4)	4 (6.5)	0.016	26 (44.8)	1 (3.5)	<0.001
Anti-HBe	2001 (54.3)	35 (56.5)	0.731	20 (34.5)	15 (17.2)	0.122
Anti-HBc	3499 (94.88)	59 (95.2)	0.999^¶^	55 (94.8)	28 (96.6)	0.999^¶^
HBV DNA, log_10_ IU/mL	2.6 (2.0-3.9)	2.0 (2.0-2,4)	<0.001	2.9 (2.0-6.5)	2.0 (2.0-2.6)	0.012
ALT, U/L	33.1 (22.1-54.5)	48.3 (31.7-69.5)	<0.001	45.9 (30.4-99.6)	41.1 (30.5-66.0)	0.763
AST, U/L	27.1 (21.3-38.1)	32.1 (23.3-44.3)	0.014	34.8 (25.3-54.7)	29.1 (22.9-39.3)	0.228
TBIL, umol/L	17.2 (13.6-21.9)	18.2 (15.1-22.1)	0.223	18.5 (16.5-23.8)	18.2 (14.6-21.9)	0.095
DBIL, umol/L	6.3 (5.3-7.9)	6.8 (6.0-8.0)	0.023	7.6 (6.3-9.1)	7.0 (6.1-7.9)	0.240
IBIL, umol/L	10.8 (8.1-14.5)	11.1 (8.9-14.5)	0.641	11.7 (9.7-14.6)	10.2 (8.5-12.8)	0.075
ALB, g/L	48.5 (46.2-50.7)	50.3 (48.1-52.0)	<0.001	49.6 (47.1-52.3)	50.4 (48.6-52.7)	0.095
GGT, U/L	23.9 (16.0-40.8)	33.4 (23.5-44.5)	<0.001	35.4 (22.4-55.0)	33.8 (23.6-43.8)	0.925
LSM, KPa	4.9 (4.0-6.5)	6.0 (4.8-7.7)	<0.001	5.7 (4.5-9.5)	6.8 (4.4-8.9)	0.653
CAP, dB/m	224 (190-264)	230 (197-267)	0.363	224 (189-272)	230 (192-260)	0.962
AFP, IU/mL	2.5 (1.4-3.9)	2.1 (1.2-3.0)	0.085	2.6 (1.3-4.1)	2.1 (1.2-2.9)	0.224

Data are presented as mean with standard deviations, median (P_25_ - P_75_) or number (%) of participants with a condition.

^¶^Fisher Exact test was performed.

AFP, alpha-fetoprotein; ALB, albumin; ALT, alanine aminotransferase; Anti-HBc, anti-Hepatitis B c antigen; Anti-HBe, anti-Hepatitis B e antigen; Anti-HBs, anti-Hepatitis B surface antigen; AST, aspartate aminotransferase; CAP, controlled decay index; DBIL, direct bilirubin; GGT, gamma-glutamyl transpeptidase; IFN, interferon; HBeAg, Hepatitis B e antigen; HBV, Hepatitis B virus; LSM, liver stiffness measurements; NUC, nucleos(t)ide-analogues; TBIL, total bilirubin.

According to a strict definition of HBsAg seroclearance, we observed 21 participants who achieved HBsAg seroclearance, corresponding to an annual incidence of 0.82 per 100 person-years. The majority of participants who achieved HBsAg seroclearance were also taking antivirals (specifically IFN), had longer HBV duration since infection, and had the highest levels of ALT, AST, DBIL, ALB and GGT, but lower level of HBV DNA (**Supplementary Table 3**).

### Characteristics of the nested case-control groups

29 participants who achieved HBsAg loss were selected and matched with 58 who had not, according to gender and age (+/- 3 years). The HBsAg loss group also had a lower proportion of HBeAg(+) cases and a lower HBV DNA load, overall. Additionally, the HBsAg loss group had a higher proportion of people who were prescribed a IFN treatment and who had an HBV vaccine history. Within this group there was also a smaller proportion who had a family history of HBV infection (**Table 1**).

Univariate logistic modelling showed that having a family history of HBV infection, taking IFN treatment, an HBV vaccine history, HBsAg level, HBV DNA level, and HBeAg status significantly associated with HBsAg loss. Multivariate-adjusted modelling with stepwise selection showed that baseline HBsAg levels negatively correlated with HBsAg loss (OR = 0.99, 95%CI = 0.98-0.99). However, IFN treatments also positively related with HBsAg loss (OR = 7.99, 95%CI = 1.62-44.88) under multivariate-adjusted modelling. Please see **Table 2** for further details.

**Table 2:** Logistic regression analysis of general features.

Variables		Univariate		Multivariate-adjusted model	
		OR (95%CI)	*p*	OR (95%CI)	*p*
Age, years	Continuous	0.99 (0.95-1.03)	0.517		
Occupation	Agricultural worker	1.00			
	Urban employee	1.36 (0.40-4.69)	0.840		
	Others	1.48 (0.54-4.03)	0.618		
Family history of HBV infection	Yes	0.37 (0.15-0.94)	0.036		
Smoking	Yes	0.93 (0.38-2.28)	0.879		
Drinking	Yes	0.55 (0.16-1.88)	0.344		
Treatment	Yes	1.66 (0.66-4.17)	0.285		
IFN	Yes	14.46 (4.15-50.48)	<0.001	7.99 (1.42-44.88)	0.018
NUC	Yes	0.60 (0.24-1.52)	0.285		
Vaccine	Yes	2.81 (1.08-7.33)	0.034		
HBV duration, years	≤10	1.00			
	>10	0.90 (0.30-2.76)	0.331		
	Unknown	2.25 (0.77-6.61)	0.088		
HBsAg, IU/mL	Continuous	0.99 (0.99-0.99)	<0.001	0.99 (0.98-0.99)	<0.001
HBV DNA, log_10_ IU/mL	Continuous	0.59 (0.41-0.86)	0.006		
HBeAg status	+	0.04 (0.01-0.35)	0.003		
ALT, U/L	Continuous	1.00 (0.99-1.00)	0.512		
AST, U/L	Continuous	1.00 (0.99-1.00)	0.493		
TBIL, umol/L	Continuous	0.94 (0.86-1.02)	0.123		
DBIL, umol/L	Continuous	0.94 (0.80-1.12)	0.504		
IBIL, umol/L	Continuous	0.90 (0.79-1.01)	0.081		
ALB, g/L	Continuous	1.14 (0.99-1.31)	0.062		
GGT, U/L	Continuous	1.00 (0.99-1.01)	0.962		
LSM, KPa	Continuous	0.99 (0.87-1.10)	0.815		
CAP, dB/m	Continuous	1.00 (0.99-1.01)	0.850		
AFP, IU/mL	Continuous	0.91 (0.78-1.05)	0.196		

AFP, alpha-fetoprotein; ALB, albumin; ALT, alanine aminotransferase; AST, aspartate aminotransferase; CAP, controlled decay index; CI, confidence interval; DBIL, direct bilirubin; GGT, gamma-glutamyl transpeptidase; HBeAg, Hepatitis B e antigen; HBV, Hepatitis B virus; IFN, interferon; LSM, liver stiffness measurements; NUC, nucleos(t)ide-analogues; OR, odds ratio; TBIL, total bilirubin.

**Figure 1:**
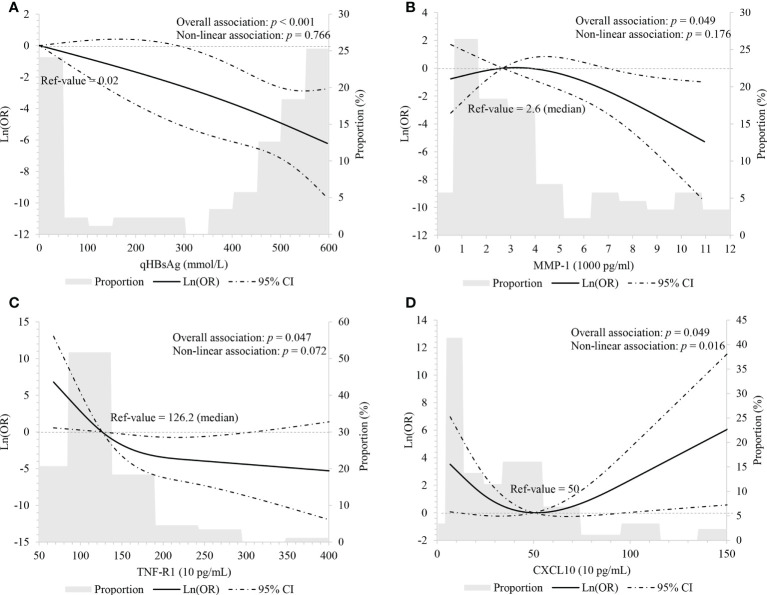
Linear association between HBsAg, cytokines and HBsAg loss using restricted cubic spline with three knots (p5, p50 and p95) **(A)** HBsAg, adjusting for age, ALT level, HBeAg status and interferon treatment; **(B-D)** MMP-1, TNF-R1, and CXCL10, adjusting for age, HBsAg level, ALT level, HBeAg status and interferon treatment.

In order to explore non-linear relationships, we performed RCS with three knots, adjusting for age, ALT level, HBeAg status, and IFN treatment intake. Results showed that baseline HBsAg levels negatively correlated with HBsAg loss in a linear manner (overall: *p *< 0.001; non-linear: *p* = 0.766, **Figure 1A**).

### Cytokines and HBsAg loss

**Figure 2:**
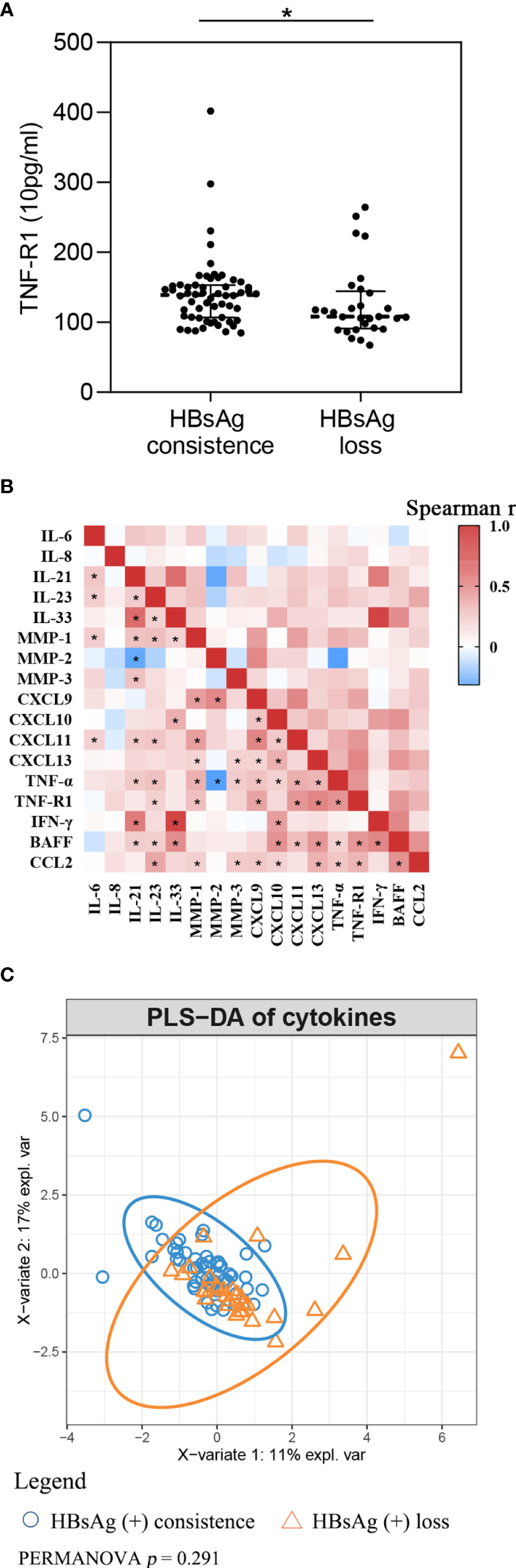
Cytokines comparation between HBsAg consistence and HBsAg loss group **(A)** TNF-R1 level between HBsAg consistence and HBsAg loss group; **(B)** Spearman correlation among cytokines. Red, means positive correlation; blue, means negative correlation; *p< 0.05 in Spearman correlation test. **(C)** Partial least squares discriminant analysis of cytokines.

Cytokine levels at baseline between HBsAg consistency and HBsAg loss were compared. Participants who achieved HBsAg loss had lower TNF-R1 (**Figure 2A** and **Supplementary Figure 2** **)**. The multivariate-adjusted logistic model showed that baseline MMP-1 (OR = 0.66, 95%CI = 0.44-0.97), CXCL9 (OR = 0.96, 95%CI = 0.93-0.99) and TNF-R1 (OR = 0.97, 95%CI = 0.94-0.99) levels negatively associated with HBsAg loss (**Table 3**).

**Table 3:** Association between cytokines and HBsAg loss.

Variables	Univariate model		Multivariate-adjusted model^§^	
	OR (95%CI)	*p*	OR (95%CI)	*p*
IL-6, pg/ml	0.99 (0.89-1.09)	0.782	0.87 (0.72-1.06)	0.175
IL-8, pg/ml	1.00 (1.00-1.00)	0.290	1.00 (1.00-1.00)	0.515
IL-21, pg/ml	1.00 (0.98-1.02)	0.900	1.00 (0.96-1.04)	0.992
IL-23, pg/ml	1.00 (0.99-1.01)	0.562	1.01 (0.98-1.03)	0.580
IL-33, pg/ml	1.01 (0.98-1.04)	0.605	0.96 (0.91-1.01)	0.085
MMP-1, 1000 pg/ml	0.91 (0.76-1.08)	0.261	0.66 (0.44-0.97)	0.036
MMP-2, 1000 pg/ml	1.07 (0.99-1.17)	0.092	0.81 (0.65-1.00)	0.055
MMP-3, 1000 pg/ml	1.01 (0.98-1.05)	0.471	1.00 (0.94-1.07)	0.968
CXCL9, 10 pg/ml	1.00 (0.98-1.01)	0.731	0.96 (0.93-0.99)	0.024
CXCL10, 10 pg/ml	1.10 (0.95-1.28)	0.197	1.00 (0.97-1.03)	0.984
CXCL11, 10 pg/ml	0.91 (0.77-1.08)	0.288	0.98 (0.95-1.01)	0.118
CXCL13, 10 pg/ml	1.09 (0.97-1.22)	0.168	1.00 (0.98-1.02)	0.634
TNF-α, 10 pg/ml	0.52 (0.17-1.61)	0.255	0.88 (0.71-1.09)	0.244
TNF-R1, 10 pg/ml	0.99 (0.98-1.00)	0.248	0.97 (0.94-0.99)	0.021
IFN-γ, 10 pg/ml	1.04 (0.96-1.12)	0.377	1.00 (0.99-1.02)	0.937
BAFF, 10 pg/ml	1.01 (0.99-1.02)	0.278	0.99 (0.97-1.00)	0.108
CCL2, 10 pg/ml	1.02 (0.98-1.05)	0.368	0.97 (0.91-1.03)	0.301

^§^Adjusting for age (continuous), HBsAg (continuous), ALT (continuous), HBeAg status (positive vs negative), and interferon treatment (yes vs no).

BAFF, B cell activating factor; CCL, C-C motif ligand; CI, confidence interval; CXCL, C-X-C motif ligand; IFN, interferon; IL, interleukin; MMP, matrix metalloproteinase; OR, odds ratio; TNF, tumor necrosis factor.

In order to assess the linearity of relations between cytokines and HBsAg loss, we performed multivariate-adjusted RCS with three knots of P5, P50 and P95. We observed baseline MMP-1 (overall: *p* = 0.049; non-linear: *p* = 0.176) and TNF-R1 (overall: *p* = 0.047; non-linear: *p* = 0.072) had decreasing associations with HBsAg loss, but this correlation appears linear (**Figures 1B, C** **)**. Baseline CXCL10 associated with HBsAg loss although the relationship appeared “U-shaped” (overall: *p* = 0.049; non-linear: *p* = 0.016). When CXCL10 < 500 pg/ml, CXCL10 had a negative relationship with HBsAg loss; and when CXCL10 ≥ 500 pg/ml, CXCL10 had an increasingly association with HBsAg loss (**Figure 1D** **)**.

As can be seen in **Figure 2B**, spearman correlation analysis suggests that cytokines highly correlate, in general. Among the cytokines, IL-21 had a strong positive association with IL-33 (r = 0.711, *p *< 0.001) and IFN-γ (r = 0.635, *p *< 0.001) at baseline, and with IL-33 with IFN-γ (r = 0.909, *p *< 0.001). We took cytokines as integral and used PLS-DA to present the global difference (**Figure 2C**). PERMANOVA with Euclidean distance was used to compare fundamental differences between the two groups. Findings suggest there is no significant difference at baseline for integral cytokine levels (*p* = 0.222).

### Cytokine SNPs and HBsAg loss

Percentages for SNPs related to cytokines have been provided in **Supplementary Figure 3**. The AT genotype for rs3806798 was significantly higher in those who encountered HBsAg loss compared to those with consistent HBsAg (**Supplementary Figure 3B**). Univariate logistic regression results showed the AT genotype of rs3806798 associated with an increased likelihood of HBsAg loss (OR = 3.52, 95%CI = 1.11-11.19). Please see **Table 4** for further details. However, after adjusting for age, HBsAg, ALT, HBeAg status, and IFN treatment, statistically significant association disappeared (**Table 4**). SE, as a measure of genetic diversity, was calculated although no difference between the groups was observed using Wilcoxon’s test (*p* = 0.713; **Supplementary Figure 3O**).

**Table 4:** Association between SNPs in relation to cytokines and HBsAg loss.

Variables		HBsAg consistence number (%)	HBsAg loss number (%)	Univariate model		Multivariate-adjusted model^§^	
				OR (95%CI)	*p*	OR (95%CI)	*p*
rs2055979	CC	16 (36.4)	12 (42.9)	1.00		1.00	
	AA/AC	28 (63.6)	16 (57.1)	0.76 (0.29-2.01)	0.582	1.08 (0.18-6.58)	0.932
rs3806798	TT	38 (86.4)	18 (64.3)	1.00		1.00	
	AA/AT	6 (13.6)	10 (35.7)	3.52 (1.11-11.19)	0.033	5.01 (0.52-48.57)	0.165
rs2069762	TT	16 (36.4)	14 (50.0)	1.00		1.00	
	GG/TG	28 (63.6)	14 (50.0)	0.57 (0.22-1.50)	0.254	0.55 (0.09-3.31)	0.521
rs1800795	GG	42 (95.5)	26 (92.9)	1.00		1.00	
	CC/CG	2 (4.5)	2 (7.1)	1.62 (0.21-12.18)	0.642	0.01 (0.01-999.99)	0.913
rs2227306	CC	16 (36.4)	11 (39.3)	1.00		1.00	
	CT/TT	28 (63.6)	17 (60.7)	0.88 (0.33-2.34)	0.803	1.13 (0.19-6.80)	0.893
rs1800469	CC	26 (59.1)	17 (60.7)	1.00		1.00	
	CT/TT	18 (40.9)	11 (39.3)	0.94 (0.36-2.46)	0.891	1.14 (0.17-7.79)	0.895
rs1800896	AA	40 (90.9)	21 (75.0)	1.00		1.00	
	GG/AG	4 (9.1)	7 (25.0)	3.33 (0.88-12.69)	0.077	9.09 (0.49-168.12)	0.138
rs1061624	GG	13 (29.5)	10 (35.7)	1.00		1.00	
	AA/AG	31 (70.5)	18 (64.3)	0.76 (0.28-2.07)	0.585	2.18 (0.25-18.89)	0.481
rs1799724	CC	32 (72.7)	21 (75.0)	1.00		1.00	
	CT/TT	12 (27.3)	7 (25.0)	0.89 (0.30-2.62)	0.831	0.72 (0.07-6.94)	0.775
rs12979860	CC	35 (79.5)	24 (85.7)	1.00		1.00	
	CT/TT	9 (20.5)	4 (14.3)	0.65 (0.18-2.35)	0.509	1.71 (0.11-26.14)	0.701
rs1799964	TT	22 (50.0)	18 (64.3)	1.00		1.00	
	CC/CT	22 (50.0)	10 (35.7)	0.56 (0.21-1.47)	0.237	0.18 (0.02-1.72)	0.135
rs1143634	CC	43 (97.7)	27 (96.4)	1.00		1.00	
	TT/CT	1 (2.3)	1 (3.6)	1.59 (0.10-26.54)	0.746	0.33 (0.01-999.99)	0.807
rs1800872	AA	23 (52.3)	11 (39.3)	1.00		1.00	
	CC/AC	21 (47.7)	17 (60.7)	1.69 (0.65-4.43)	0.284	4.40 (0.59-33.01)	0.150
rs3025058	6A6A	34 (77.3)	18 (64.3)	1.00		1.00	
	5A5A/5A6A	10 (22.7)	10 (35.7)	1.89 (0.66-5.38)	0.234	1.74 (0.22-13.77)	0.600

^§^Adjusting for age (continuous), HBsAg (continuous), ALT (continuous), HBeAg status (positive vs negative), and interferon treatment (yes vs no).

CI, confidence interval; OR, odds ratio; SNP, single nucleotide polymorphism.

## Discussion

In this cohort study of HBV patients from Wuwei city, 2.41% encountered HBsAg loss and 0.82% achieved HBsAg seroclearance. In this instance, both scenarios were either spontaneous or initiated through antiviral treatments. During the nested case-control analysis of HBsAg carriers, lower HBsAg levels at baseline and IFN treatment were associated with an increased likelihood of encountering HBsAg loss. In addition, participants with lower levels of MMP-1, CXCL9 and TNF-R1 at baseline appear more likely to achieve HBsAg loss. Baseline CXCL10 appears to be associated with HBsAg loss although the relationship appears to be “U-shaped”.

We found that lower HBsAg levels at baseline is associated with an increased likelihood of encountering HBsAg loss. Lower HBsAg levels at baseline also appear to hold the greatest predictive value for HBsAg seroclearance, as participants with lower HBsAg levels achieve HBsAg loss easier and sooner (19, 20). We also found that IFN treatment was associated with an increased likelihood of HBsAg loss which may be indicative of other outcomes. A meta-analysis based on six clinical controlled trials recently reported that IFN could increase the likelihood of HBsAg seroclearance (21). In addition, IFN-based therapy can lead to more significant HBsAg reduction compared to therapies based on nucleoside and/or nucleotide analogues, leading to an increased likelihood of HBsAg seroclearance (22, 23). This provides evidence to support the use of IFN with (or without) NUC for both naïve and NUC-exposed patients (23). Although, local and regional experience tells us that the use of IFN therapies in the north-west of China remains uncommon. This is something which could be addressed relatively quickly with training or by updating the current Chinese guidelines.

A recent study found that baseline CXCL10/IP10 could be used to predict HBsAg decline in HBeAg-negative patients receiving Entecavir. This is because patients with baseline IP10 > 350 pg/ml were also found to achieve significantly more rapid HBsAg decline i.e. ≥ 0.5 log10 (HR = 4.39, 95%CI = 1.63-11.83) (24). Likewise, a cross-sectional study observed higher level of IP10 in CHB patients with HBsAg seroclearance (25). However, Wong et al. found that lower serum IP-10 levels at year zero (i.e. the time of achieving HBsAg seroclearance) is the only cytokine associated with HBsAg seroclearance (HR per 100 pg/mL = 0.82, 95% CI 0.85-0.99) (14).

Interestingly, we observed that CXCL10 was associated with HBsAg loss and this relationship appears “U-shaped” with the lowest point in the trough occurring at 500 pg/mL. That is to say, the plot highlights a substantial reduction in risk within the lower range of CXCL10, which is consistent with previous studies which have reported the lowest risk of around 500 pg/mL followed by an increase. However, the number of participants in our study was relatively small, especially for those with higher IP10 levels, therefore our study provides only a potential explanation for the aforementioned inconsistencies. Secondly, research suggests that CXCL10 declines before increasing over the treatment period (14, 24). Therefore, CXCL10 levels taken at different times may also become an important measure for assessing relationships.

We also observed an association between lower levels of plasma CXCL9 at baseline and a higher likelihood of HBsAg loss in CHB patients. Similar results have only been found in one acute hepatitis study, where CXCL9, CXCL10, CXCL11 and CXCL13 elevated during the acute phase but then decreased in conjunction with an HBsAg decline (26). However, some researchers have suggested this is not the case for CHB patients (26, 27). Such inconsistent results may be due to differences between study populations, limited sample sizes and variations in outcome definitions. Although, CXCL9 and CXCL10, also known as MIG and IP-10, are all members of CXCL family and could promote Th1 responses as well as initiate CD8+ and natural killer (NK) cell trafficking (28, 29). Increased NK cell functions and HBV-specific CD8+ T cell responses are also associated with HBsAg seroclearance (30). This of course requires further basic medical research but could be relatively easily confirmed.

MMP-1 is one metalloproteinase that has a role in both degrading and denaturing interstitial collagens, types I, II and III. In this study, we observed an association between lower levels of plasma MMP-1 and a greater likelihood of HBsAg loss, which has not yet been reported. On the other hand, the positive relationship between MMP-1 and adverse outcomes for CHB patients has been reported. For example, Flisiak et al. found that MMP-1 levels continue to rise during the first four weeks of acute viral hepatitis, which seems connected to hepatocytes damage (31). Yang et al. (32) also found that MMP-1 significantly increases in HCC patients and could therefore be useful biomarkers for the early HCC detection and in prognostics. Although, the role of MMP-1 during HBsAg loss is not fully understood and requires further investigation.

TNF-R1 appears on almost all cell types and mediates inflammatory responses, cytotoxic action, antiviral activity, and cell proliferation (33). Yang et al. (34) found that TNF-R1 is required to eliminate the natural cccDNA transcriptional template during natural HBV infection. Likewise, Tai et al. (35) observed soluble TNF-R1 levels are higher in CHB patients compared to healthy controls, and that TNF-R1 levels correlate with liver inflammation in CHB cases. In our study, we observed a negative association between plasma TNF-R1 at baseline and the likelihood of HBsAg loss. This may support the theory that participants with reduced inflammation are more likely to lose HBsAg although again further research is required. 

In this study, we assumed that participants with HBsAg loss were more likely to present with an anti-inflammatory mode of cytokines, characterized by lower levels of MMP-1, CXCL9, CXCL10 and TNF-R1 at baseline. Therefore, we performed PLS-DA analysis to compare the fundamental differences between the two groups. We did not observe any significant differences although we did manage to develop a more comprehensive description of cytokines.

Before making any recommendations, it is important to first discuss the limitations involved. Firstly, we choose HBsAg loss rather than seroclearance as the main outcome of interest. One reason for this was that our cohort was based on only three years and 71.1% were followed less than twice. This meant we were unable to meet follow-up time criteria for HBsAg seroclearance. We also chose HBsAg loss hoping to gain insight into how HBsAg seroclearance is achieved. However, we accept that we still need to develop our understanding of the relationship between HBsAg loss and HBsAg seroclearance. Secondly, we only observed 62 participants who had achieved HBsAg loss and among these only 29 had complete data. This sample is small however we implemented a matching process (ratio 1:2) to increase inspection efficiency. Finally, we only examined the association between baseline characteristics and HBsAg loss. Some characteristics are time-specific and vary during follow-up. This variability may also influence HBsAg loss although further research is required.

## Conclusion

This was a nested case-control study of HBsAg carriers from a cohort in Wuwei city, China. Lower levels of HBsAg, MMP-1, CXCL9 and TNF-R1 at baseline and IFN treatment were associated with an increased likelihood of HBsAg loss. Baseline CXCL10 also appears associated with HBsAg loss but the relationship appears U-shaped, which requires further investigation. It may be possible to develop a nomogram which intercalates these factors; however, researchers should consider immune processes involved in HBsAg loss. In order to improve interventions efficacy we should pay attention to immune activities related HBsAg loss.

## Data availability statement

The datasets used in this study can be found in the **Supplementary Material**.

## Ethics statement

The studies involving human participants were reviewed and approved by The Ethics Committee of the Air Force Medical University. The patients/participants provided their written informed consent to participate in this study.

## Author contributions

ZS and WlZ designed the study, reviewed, and revised the manuscript. XY and TF analyzed the data and wrote the manuscript. LX, ZH, HZ, XK, SZ, QZ and YL did laboratory tests. ZJ and SS revised the manuscript and helped to interpret the results. WhZ and YY managed the cohort. WJ, CL, HT and FW collected data. All authors read and approved the final manuscript.

## Funding

This study was supported by China Special Grant for the Prevention and Control of Infection Diseases (2017ZX10105011), National Natural Science Foundation of China (81773488), and the Natural Science Foundation of Shaanxi Province (2021JQ-341).

## Acknowledgments

The authors thank the participants who agreed to participate and made this cohort study possible.

## Conflict of interest

The authors declare that the research was conducted in the absence of any commercial or financial relationships that could be construed as a potential conflict of interest.

## Publisher’s note

All claims expressed in this article are solely those of the authors and do not necessarily represent those of their affiliated organizations, or those of the publisher, the editors and the reviewers. Any product that may be evaluated in this article, or claim that may be made by its manufacturer, is not guaranteed or endorsed by the publisher.

## Glossary

**Table:**

AFP	alpha fetoprotein
AH	acute hepatitis
ALT	alanine aminotransferase
ALB	albumin
AST	aspartate aminotransferase
BAFF	B cell activating factor
CAP	controlled decay index
CI	confidence interval
CCL	C-C motif ligand
CHB	chronic hepatitis B
CXCL	C-X-C motif ligand
DBIL	direct bilirubin
ELISA	enzyme-linked immunosorbent assay
GGT	gamma-glutamyl transpeptidase
HBeAg	hepatitis B e antigen
HBsAg	hepatitis B surface antigen
HBV	hepatitis B virus
HCC	hepatocellular carcinoma
IBIL	indirect bilirubin
IFN	interferon
IL	interleukins
IP	interferon-inducible protein
LSM	liver stiffness measurements
MICE	multivariate imputation by chained equations
miR	microRNAs
MMP	matrix metalloproteinases
NK	natural killer
OR	odds ratio
PERMANOVA	permutational multivariate analysis of variance
PLS-DA	partial least squares discriminant analysis
PYs	person-years
RCS	restricted cubic spline
RT-qPCR	reverse transcription quantitative polymerase chain reaction
SD	standard deviation
SE	shannon entropy
SNP	single nucleotide polymorphisms
TBIL	total bilirubin
TGF	transforming growth factor
TNF	tumor necrosis factor
