# Supplementary material for: Describing immune factors associated with Hepatitis B surface antigen loss: A nested case-control study of a Chinese sample from Wuwei City
Source: Front Immunol. 2022 Oct 11;13:1025654. doi: 10.3389/fimmu.2022.1025654 (PMC9592898; doi:10.3389/fimmu.2022.1025654)
Supplement: Supplementary file 2 [file DataSheet_2.docx]

**SUPPLEMENTARY MATERIALS**

**Supplementary Methods**

**Supplementary Results**

**Supplementary Discussion**

**Supplementary References**

**Supplementary Figure 1.** Work-flow diagram

**Supplementary Figure 2.** Cytokines level at baseline in the HBsAg consistence and HBsAg loss groups

**Supplementary Figure 3.** Percentage of subtypes of human SNPs in the HBsAg consistence and loss groups

**Supplementary Figure 4.** MicroRNAs level at baseline in the HBsAg consistence and HBsAg loss groups

**Supplementary Table 1.** Primers of 14 tested cytokine SNPs for PCR

**Supplementary Table 2.** Missing Rate

**Supplementary Table 3.** Baseline characteristics of the HBsAg consistence, HBsAg loss but not seroclearance and the HBsAg seroclearance groups

**Supplementary Table 4.** Association between features and HBsAg loss using RCS with three knots

**Supplementary Table 5.** Association between microRNAs and HBsAg loss

**Supplementary Table 6.** Feature changes

**Methods**

**Examinations in Gansu Wuwei Tumor Hospital**

Liver function tests including aspartate aminotransferase (AST), alanine aminotransferase (ALT), total bilirubin (TBIL), direct bilirubin (DBIL), indirect bilirubin (IBIL), gamma-glutamyl transpeptidase (GGT) and albumin (ALB) concentrations were measured using standard laboratory tests. A chemiluminescence analysis reagent (Roche, Mannheim, Germany) was used for assessing alpha fetoprotein (AFP).

Enzyme-linked immunosorbent assay (ELISA) reagents (KHB, Shanghai, China) were used to initially test HBsAg, HBeAg, anti-HBs, anti-HBe, anti-HBc and for anti-hepatitis B virus. Serum HBV DNA levels were measured using a hybrid capture assay (DAAN gene, Guangzhou, China) before 2019, and then, by using a PCR-fluorescence probing assay (ABBOTT, Des Plaines, USA).

A one-dimensional transient elastography technique was applied with the Fibroscan^®^ (Echosens, Paris, France) system and was used in the controlled decay index (CAP) and for liver stiffness measurements (LSM).

**Cytokines**

All previously frozen serum samples required centrifugation at 16,000 g for four minutes immediately prior to dilution. Supernatants required a 2-fold dilution (75ul of sample + 75ul of calibrator duliuent RD6-52). Standards were prepared through multiple dilution in accordance with RD-kit requirements, producing a 3-fold dilution series. Samples were prepared in accordance with RD-kit instructions, and were analyzed within 90 minutes. The Certificate of Analysis listed our standardized concentrations and 3-fold dilutions were calculated for each remaining level.

**SNPs**

Universal reaction conditions for each genotype were as follows: 5μl DNA, 2μl primer and probe premix with 10μl RealFAST Probe PCR mix were made to a final volume of 25μl with 8μl of double distilled water. The PCR cycle consisted of an initial denaturation at 95℃ for 5 minutes followed by 40 cycles at 95℃ for 10 seconds and 60℃ for 30 seconds. Primers for 14 cytokine SNPs have been presented in the **Supplementary Table 1**.

**MicroRNAs**

Six microRNAs (miR), including miR-375, miR-92a, miR-500a, miR-155, miR-146 and miR-192, were isolated from whole blood samples using TRIzol reagent (Invitrogen, Carlsbad, USA). These were quantified through reverse transcription quantitative polymerase chain reaction (RT-qPCR) using TB Green® Premix Ex Taq™ II (TAKARA, Dalian, China) according to the manufacturer’s instructions. U6 was used as an endogenous control and the relative expression was analyzed using the 2^-∆∆Ct^ method (1).

**Statistical analysis**

Several LSM, CAP and AFP levels were missing because of rejection but were imputed using Multivariate Imputation by Chained Equations (MICE) which requires “proc MI” in SAS. SNPs from 15 participants and microRNAs from 18 participants were missing due to blood sample volume limitations. Missing rates are also presented in **Supplementary Table 2**.

The “%RCS_REG” microprogram developed by Desquilbet was applied to assess the linearity of relationships (2). A restricted cubic spline (RCS) model was first built with five knots (i.e. p5, p25, p50, p75, and p95) for flexible descriptions of non-linearity. P values for splines 4, 3, 2, and 1, linear 0 was checked sequentially. When *p* values were not below the 0.05 threshold, the places for the five knots were adjusted to better fit the linearity of the relationship. If this adjustment did not improve fit, the number of knots was reduced to four (i.e. p5, p25, p75, and p95) and actions (as previously described) were repeated. If these adjustments still did not improve fit, knots were again reduced to three (i.e. p5, p50, and p95). If *p* values for splines 1 and 2 as well as the *p* value for non-linear associations with three knots were non-significant, associations were considered linear.

**Results**

**MicroRNAs and HBsAg loss**

We did not observe any difference between participants who achieved HBsAg loss or in those not on miR-375, miR-92a, miR-500a, miR-155, miR-146 and miR-192 (**Supplementary Figures 4A-F**). No relationship between microRNAs and HBsAg loss was observed in either the logistic regression model, RCS model or through PLS-DA (**Supplementary Tables 2, 5 and Supplementary Figure 4G**).

**Change of Liver function,** **Cytokines, MicroRNAs and HBsAg loss**

Variable changes were calculated as: change = afterwards - baseline, and were analyzed using a single sample Wilcoxon’s Signed Rank test. Both groups showed decreased levels of HBsAg, HBV DNA, TBIL, DBIL, ALB and increased levels of AFP, IL-23, TNF-α and miR-155. In addition, the HBsAg loss group had a lower level of LSM while the HBsAg consistence group had lower levels of ALT, GGT, IL-21, IFN-γ but a higher level of miR-500a. IL-33 decreased more in the HBsAg consistence group compared to the HBsAg loss group. Please see **Supplementary Table 6** for further details.

**Sensitivity Analysis**

One of our 29 HBsAg loss participants experienced serological reversion. After excluding this participant, our results remained unchanged. When we implemented a strict definition of HBsAg seroclearance, only twelve participants achieved HBsAg seroclearance. When we repeated multivariate-adjusted logistic analyses using cytokines, SNPs and microRNAs, no significant associations were observed (data not shown).

**Discussion**

A recent meta-analysis reported a pooled annual HBsAg seroclearance rate of 1.02% which were either spontaneous or the result of antiviral treatment (3). In the Asia Pacific region, this proportion is 1.09% which is comparatively higher than other regions (3). In China, the HBsAg seroclearance appears markedly higher with 1.80% in CHB patients (4). Stricter policies, combined injections for mothers with CHB, and further investment in prevention, are likely to be contributory factors. However, not enough is known about specific regions of China. Evidence suggests the incidence in the south-east of China ranges from 0.54% to 3.31% (5-8) which suggests public health interventions need to be more highly contextualised. We observed an HBsAg seroclearance rate of only 0.82%, although this may due to the relatively short follow-up time of 1.5 years. Also, 71.1% of our sample were followed fewer than two times which may be insufficient for HBsAg seroclearance. Further research into specific regions of China is called for, in conjunction with the ongoing cohort study in Wuwei City.

We further explored the SNPs related to cytokines with HBsAg loss, including IL-1B, IL-2, IL-6, IL-8, IL-10, IL-15, IL-21, IL-28B, transforming growth factor (TGF)-β1, TNF, TNF-α, TNF-R2 and MMP-3. Only the AT genotype of rs3806798 was observed to associate with an increased likelihood of HBsAg loss (OR = 3.52, 95%CI = 1.11-11.19) in the univariate logistic model. Previous studies of the IL-6 gene (rs1800795) have also observed an association with HBV infection (9). However, in this study we did not observe an association between rs1800795 and HBsAg loss. No study has previously reported this association and therefore our negative results appear consistent.

**References**

1. Schmittgen TD, Livak KJ. Analyzing real-time PCR data by the comparative C(T) method. Nat Protoc 2008;3:1101-1108.

2. Desquilbet L, Mariotti F. Dose-response analyses using restricted cubic spline functions in public health research. Stat Med 2010;29:1037-1057.

3. Yeo Y, Ho H, Yang H, Tseng T, Hosaka T, Trinh H, Kwak M, et al. Factors Associated With Rates of HBsAg Seroclearance in Adults With Chronic HBV Infection: A Systematic Review and Meta-analysis. Gastroenterology 2019;156:635-646.e639.

4. Zu J, Zhuang G, Liang P, Cui F, Wang F, Zheng H, Liang X. Estimating age-related incidence of HBsAg seroclearance in chronic hepatitis B virus infections of China by using a dynamic compartmental model. Scientific reports 2017;7:2912.

5. Liu J, Yang H, Lee M, Lu S, Jen C, Wang L, You S, et al. Incidence and determinants of spontaneous hepatitis B surface antigen seroclearance: a community-based follow-up study. Gastroenterology 2010;139:474-482.

6. Zhu L, Zhai X, Wang Q, Jiang J, Peng H, Song C, Ge Z, et al. Incidence and determinants of spontaneous hepatitis B surface antigen seroclearance and seroconversion in hepatitis B e antigen-negative chronic infection patients: A population-based prospective cohort. Journal of viral hepatitis 2018;25:1588-1598.

7. Han ZG, Qie ZH, Qiao WZ. HBsAg spontaneous seroclearance in a cohort of HBeAg-seronegative patients with chronic hepatitis B virus infection. J Med Virol 2016;88:79-85.

8. Chen Q, Wang X, Harrison T, He X, Hu L, Li K, Jia H, et al. HBsAg may reappear following reactivation in individuals with spontaneous HBsAg seroclearance 8 years previously. Epidemiology and infection 2017;145:728-738.

9. Riazalhosseini B, Mohamed Z, Apalasamy YD, Shafie NS, Mohamed R. Interleukin-6 gene variants are associated with reduced risk of chronicity in hepatitis B virus infection in a Malaysian population. Biomed Rep 2018;9:213-220.

**Supplementary Figure 1.** Work-flow diagram

HBsAg

MicroRNA

Chemiluminescence

qRCR

Cytokines

Liquid chips

Blood Sample Test

Human

SNPs

qRCR

**Nested case-control study**

**(n = 87)**

Clinical Metadata

**Factors of HBsAg loss**

Questionnaire

**Cohort**

**(n = 3,750)**

Excluding:

1. Lacking blood samples for quantitative HBsAg test (n = 25);
2. Inconsistence result between qualitative and quantitative HBsAg (n = 8);

HBsAg (+) loss

(n = 62)

HBsAg (+) consistence

(n = 58)

HBsAg (+) loss

(n = 29)

Gender

Age (+/- 3)


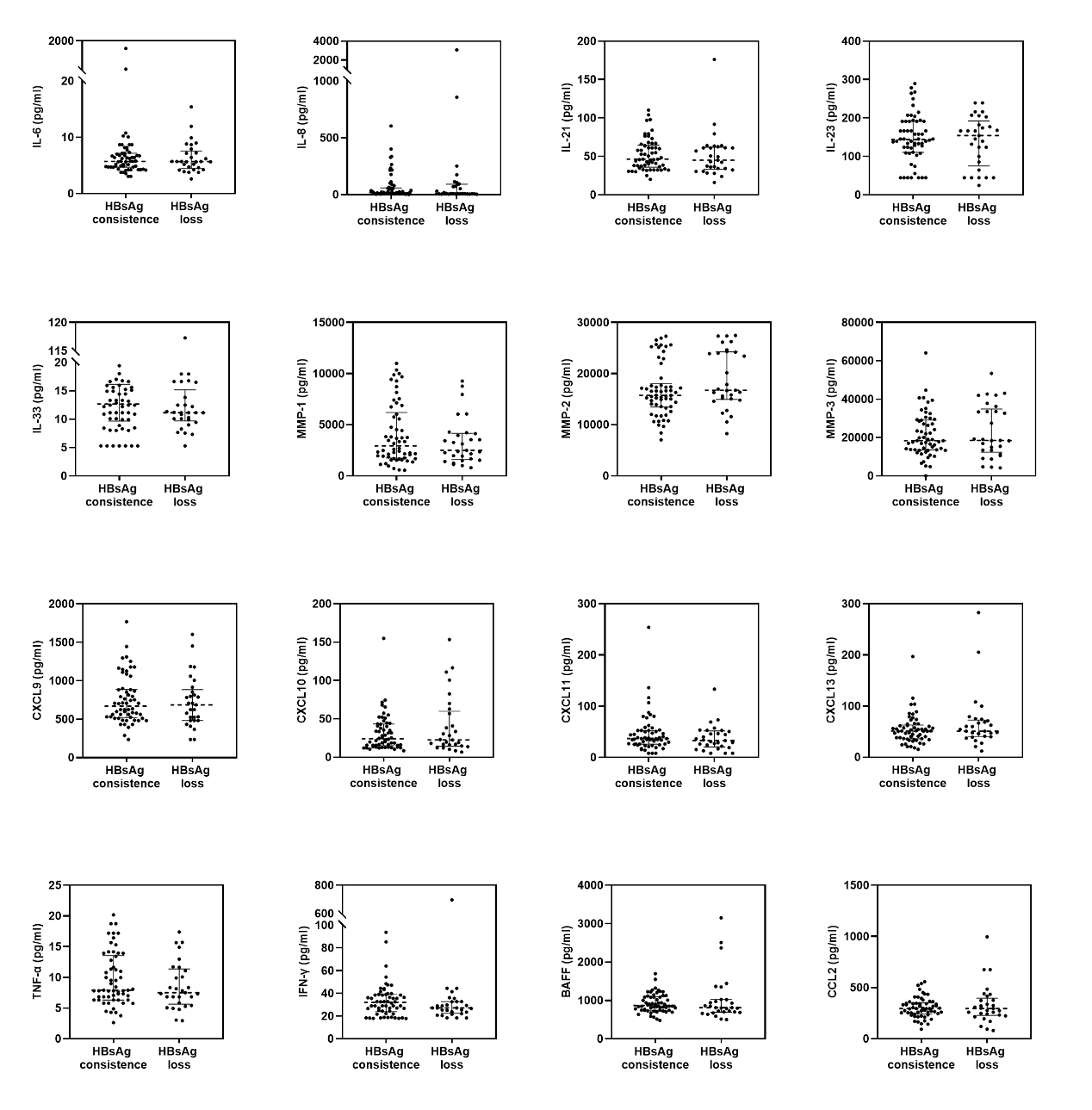


**Supplementary Figure 2.** Cytokine levels at baseline for the HBsAg consistence and HBsAg loss groups

**Supplementary Figure 3.** Percentage of subtypes for human single nucleotide polymorphisms in the HBsAg (+) consistence and HBsAg loss groups

**
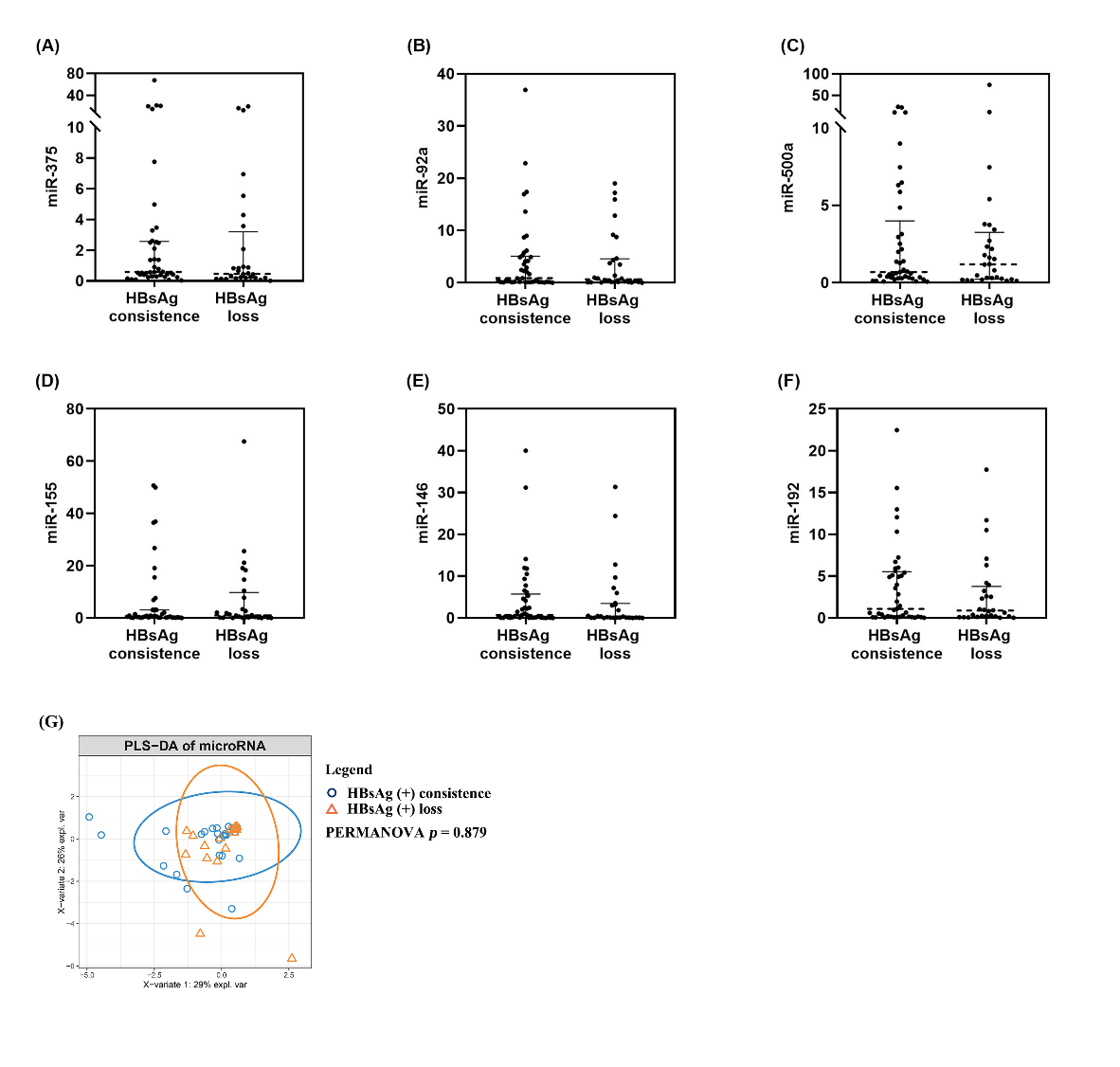
Supplementary Figure 4.** MicroRNAs levels at baseline for the HBsAg consistence and HBsAg loss group

**Supplementary Table 1.** Primers and probes for 14 tested SNPs in relation to cytokines

|  | Primer | Probe | Allele |
| --- | --- | --- | --- |
| rs2055979 | |  |  |
| Forward | AGCACATTCAGCTTATTGGAAAGA | ATTTCTAGCATCTCATTCA | T |
| Reverse | GGAAACTCTGGAAAGAACTCTAACCA | ATTTCTAGCATCTCATGCA | G |
| rs3806798^#^ | |  |  |
| Forward | GCACAGGCTGCAAGAAGGA | CTTTGATGTCCAACTAG | T |
| Reverse | GGGTGTCTTCTTCCAACATTTCC | CTTTGAAGTCCAACTAG | A |
| rs2069762 | |  |  |
| Forward | TCCACCACAATATGCTATTCACATG | CAGTGTAGTTTTATGACAAA | T |
| Reverse | CGCCTTCTGTATGAAACAGTTTTTC | TCAGTGTAGTTTTAGGACAA | G |
| rs1800795^#^ | |  |  |
| Forward | GATTGTGCAATGTGACGTCCTT | AGCATGGCAAGACA | G |
| Reverse | GCTGCACTTTTCCCCCTAGTT | AGCATCGCAAGACA | C |
| rs2227306^#^ | |  |  |
| Forward | CCTAGCCCTTGACCTCAGTTAGTT | TGACAACATTGAACGAC | G |
| Reverse | ACCATGAAGATGTTGATATTGTACAAAAAG | AACATTGAACAACTTC | A |
| rs1800469 | |  |  |
| Forward | TGGGAGGTGCTCAGTAAAGGA | CCTTCCATCCTTCA | T |
| Reverse | GGTAGGAGAAGAGGGTCTGTCAAC | CCCTTCCATCCCTCAG | C |
| rs1800896^#^ | |  |  |
| Forward | GGTCCCTTACTTTCCTCTTACCTATCC | TACTTCCCCTTCCCAAA | T |
| Reverse | ACACACAAATCCAAGACAACACTACTAA | TACTTCCCCCTCCC | C |
| rs1061624^#^ | |  |  |
| Forward | CCATGGCAGCAGAGGCTTT | CACAACTCGCTGCC | T |
| Reverse | TTCTGGGCCAAGTTCCTCTAGT | ACAACCCGCTGCC | C |
| rs1799724 | |  |  |
| Forward | GGAGAATGTCCAGGGCTATGG | CCCCCCCTTAATGA | T |
| Reverse | TGGAGGTCCTGGAGGCTCTT | ACCCCCCCTTAACGA | C |
| rs12979860 | |  |  |
| Forward | TGCCTGTCGTGTACTGAACCA | TCCCCGAAGGCGTGA | T |
| Reverse | GAGCGCGGAGTGCAATTC | CGAAGGCGCGAAC | C |
| rs1799964 | |  |  |
| Forward | GGTGAGGCCGCCAGACT | AGAAGATGAAGGAAAAG | T |
| Reverse | TCTCCTGTAACCCATTCCTCAGA | AAGCTGAGAAGACGAA | C |
| rs1143634 | |  |  |
| Forward | GCCTGCCCTTCTGATTTTATACC | TTCAGAACCTATCTTCTTTGA | T |
| Reverse | CGTGCACATAAGCCTCGTTATC | TCAGAACCTATCTTCTTCGA | C |
| rs1800872 | |  |  |
| Forward | TGTGCCTGAGAATCCTAATGAAATC | CCGCCTGTCCTGTAG | C |
| Reverse | AAGCAGCCCTTCCATTTTACTTT | ACCCCGCCTGTACT | A |
| rs3025058^#^ | |  |  |
| Forward | TTCTCCTTGTCCTCATATCAATGTG | AAGACATGGTTTTTTC | T |
| Reverse | AGTATTCTATGGTTCTCCATTCCTTTG | AGACATGGTTTTTCC | - |

^#^ Probe was designed in reverse sequence.

**Supplementary Table 2.** Missing rate of variables

| **Variables** | **Missing Number** | **Missing Rate** |
| --- | --- | --- |
| LSM | 3 | 3.4% |
| CAP | 3 | 3.4% |
| AFP | 1 | 1.1% |
| rs2055979 | 15 | 17.2% |
| rs3806798 | 15 | 17.2% |
| rs2069762 | 15 | 17.2% |
| rs1800795 | 15 | 17.2% |
| rs2227306 | 15 | 17.2% |
| rs1800469 | 15 | 17.2% |
| rs1800896 | 15 | 17.2% |
| rs1061624 | 15 | 17.2% |
| rs1799724 | 15 | 17.2% |
| rs12979860 | 15 | 17.2% |
| rs1799964 | 15 | 17.2% |
| rs1143634 | 15 | 17.2% |
| rs1800872 | 15 | 17.2% |
| rs3025058 | 15 | 17.2% |
| miR-375 | 18 | 20.7% |
| miR-92a | 18 | 20.7% |
| miR-500a | 18 | 20.7% |
| miR-155 | 18 | 20.7% |
| miR-146 | 18 | 20.7% |
| miR-192 | 18 | 20.7% |

**Supplementary Table 3.** Baseline characteristics for the HBsAg consistence, HBsAg loss not seroclearance, and HBsAg seroclearance groups

| **Variables** | **HBsAg consistence (N=3,688)** | **HBsAg seroclearance (n=21)** | **HBsAg loss not seroclearance (n=41)** | ***p*** |
| --- | --- | --- | --- | --- |
| Age, years | 46.2±12.9 | 47.6±10.0 | 47.2±11.9 | 0.779 |
| Gender |  |  |  | **0.024** |
| Male | 2160 (58.6) | 16 (76.2) | 31 (75.6) |  |
| Female | 1528 (41.4) | 5 (23.8) | 10 (24.4) |  |
| Occupation |  |  |  | 0.082 |
| Agricultural labourer | 1771 (48.0) | 8 (38.1) | 19 (46.3) |  |
| Urban employee | 562 (15.2) | 5 (23.8) | 12 (29.3) |  |
| Others | 1355 (36.7) | 8 (38.1) | 10 (24.4) |  |
| Smoker | 1279 (34.7) | 12 (57.1) | 14 (34.2) | 0.098 |
| Drinker | 570 (15.5) | 5 (23.8) | 8 (19.5) | 0.449 |
| Antiviral treatment |  |  |  | **<0.001^¶^** |
| NUC | 871 (23.6) | 5 (23.8) | 7 (17.1) |  |
| IFN | 206 (5.6) | 8 (38.1) | 7 (17.1) |  |
| Both | 100 (2.7) | 3 (14.3) | 6 (14.6) |  |
| None | 2511 (68.1) | 5 (23.8) | 21 (51.2) |  |
| IFN | 306 (8.3) | 11 (52.4) | 13 (31.7) | **<0.001** |
| NUC | 971 (26.3) | 8 (38.1) | 13 (31.7) | 0.426 |
| Vaccine | 821 (22.3) | 6 (28.6) | 11 (26.8) | 0.619 |
| Cirrhosis | 220 (6.0) | 2 (9.5) | 4 (4.9) | 0.673^¶^ |
| Family history of HBV | 1853 (50.2) | 8 (38.1) | 20 (48.8) | 0.531 |
| HBV Course |  |  |  | **<0.001** |
| ≤10years | 811 (22.0) | 7 (33.3) | 17 (41.5) |  |
| >10 years | 538 (14.6) | 8 (38.1) | 10 (24.4) |  |
| Unknown | 2339 (63.4) | 6 (28.6) | 14 (34.2) |  |
| Anti-HBs | 31 (0.8) | 0 (0.0) | 1 (2.4) | 0.495^¶^ |
| HBeAg | 678 (18.4) | 0 (0.0) | 4 (9.8) | **0.035** |
| Anti-HBe | 2001 (54.3) | 12 (57.1) | 23 (56.1) | 0.940 |
| Anti-HBc | 3499 (94.9) | 20 (95.2) | 39 (95.1) | 0.999^¶^ |
| HBV DNA, log_10_ IU/mL | 2.6 (2.0-3.9) | 2.0 (2.0-2.2) | 2.0 (2.0-2.6) | **<0.001** |
| ALT, U/L | 33.1 (22.1-54.5) | 55.3 (30.0-85.2) | 46.9 (33.4-64.3) | **<0.001** |
| AST, U/L | 27.1(21.3-38.1) | 34.0 (26.0-69.6) | 31.0 (22.9-42.0) | **0.029** |
| TBIL, umol/L | 17.2(13.6-21.9) | 18.7 (15.2-22.9) | 17.6 (14.8-21.9) | 0.399 |
| DBIL, umol/L | 6.3(5.3-7.9) | 7.3 (6.1-8.5) | 6.7 (5.8-7.9) | **0.048** |
| IBIL, umol/L | 10.8(8.1-14.5) | 12.1 (9.0-14.5) | 10.9 (8.9-14.4) | 0.881 |
| ALB, g/L | 48.5(46.2-50.7) | 50.6 (48.2-52.0) | 50.2 (48.1-52.0) | **<0.001** |
| GGT, U/L | 23.9(16.0-40.8) | 44.5 (25.7-63.1) | 33.3 (23.5-38.6) | **0.002** |
| LSM, KPa | 4.9(4.0-6.5) | 5.7 (4.6-11.7) | 6.4 (4.8-7.7) | **0.004** |
| CAP, dB/m | 224(190-264) | 225 (181-257) | 230 (211-270) | 0.345 |
| AFP, IU/mL | 2.5(1.4-3.9) | 2.7 (1.5-3.5) | 2.1 (1.1-2.6) | 0.092 |

**NB*** Data are presented as mean ± standard deviation, median (P_25_ - P_75_) or number (%) of participants with a condition. ^¶^ Fisher Exact test was performed.

**Abbreviations**: AFP, alpha-fetoprotein; ALB, albumin; ALT, alanine aminotransferase; Anti-HBc, anti-Hepatitis B c antigen; Anti-HBe, anti-Hepatitis B e antigen; Anti-HBs, anti-Hepatitis B surface antigen; AST, aspartate aminotransferase; CAP, controlled decay index; DBIL, direct bilirubin; GGT, gamma-glutamyl transpeptidase; IFN, interferon; HBeAg, Hepatitis B e antigen; HBV, Hepatitis B virus; LSM, liver stiffness measurements; NUC, nucleos(t)ide-analogues; TBIL, total bilirubin

**Supplementary Table 4.** Associations between features and HBsAg loss using RCS with three knots^§^

| **Variables** | **Overall association** | | **Non-linear association** | |
| --- | --- | --- | --- | --- |
|  | **Wald** | ***p*** | **Wald** | ***p*** |
| Age | 1.03 | 0.591 | 0.45 | 0.502 |
| ALT | 2.89 | 0.236 | 0.12 | 0.733 |
| AST | 2.36 | 0.307 | 1.92 | 0.166 |
| TBIL | 2.83 | 0.243 | 1.38 | 0.240 |
| DBIL | 1.32 | 0.518 | 0.72 | 0.396 |
| IBIL | 3.01 | 0.222 | 1.79 | 0.181 |
| ALB | 2.19 | 0.335 | 0.24 | 0.628 |
| GGT | 0.17 | 0.919 | 0.04 | 0.844 |
| LSM | 5.08 | 0.079 | 3.11 | 0.078 |
| CAP | 3.41 | 0.182 | 3.41 | 0.065 |
| AFP | 5.05 | 0.080 | 4.81 | 0.028 |
| IL-6 | 2.37 | 0.305 | 0.09 | 0.766 |
| IL-8 | 3.25 | 0.197 | 3.09 | 0.079 |
| IL-21 | 0.03 | 0.987 | 0.03 | 0.873 |
| IL-23 | 3.38 | 0.185 | 3.04 | 0.811 |
| IL-33 | 3.37 | 0.186 | 0.85 | 0.357 |
| MMP-2 | 3.71 | 0.156 | 0.01 | 0.911 |
| MMP-3 | 2.73 | 0.255 | 2.73 | 0.098 |
| CXCL9 | 5.22 | 0.074 | 0.13 | 0.716 |
| CXCL11 | 2.71 | 0.258 | 0.02 | 0.883 |
| CXCL13 | 0.24 | 0.886 | 0.01 | 0.928 |
| TNF-α | 1.66 | 0.437 | 0.29 | 0.588 |
| IFN-γ | 0.01 | 0.996 | 0.01 | 0.976 |
| BAFF | 3.06 | 0.217 | 1.21 | 0.271 |
| CCL2 | 2.25 | 0.325 | 1.28 | 0.257 |
| miR375 | 1.49 | 0.474 | 0.19 | 0.660 |
| miR92a | 2.89 | 0.236 | 1.01 | 0.314 |
| miR500a | 0.11 | 0.944 | 0.11 | 0.735 |
| miR155 | 1.35 | 0.508 | 0.02 | 0.888 |
| miR146 | - | - | - | - |
| miR192 | 1.61 | 0.448 | 0.61 | 0.435 |

^§^ Adjusting for age (continuous), HBsAg (continuous), ALT (continuous), HBeAg status (positive vs negative), and interferon treatment (yes vs no).

**Supplementary Table 5.** Associations between microRNAs and HBsAg loss

| **Variables** | **Univariate model** | | **Multivariate-adjusted model^§^** | |
| --- | --- | --- | --- | --- |
|  | **OR (95%CI)** | ***p*** | **OR (95%CI)** | ***p*** |
| miR375 | 0.98 (0.91-1.04) | 0.456 | 1.08 (0.96-1.21) | 0.188 |
| miR92a | 0.99 (0.92-1.06) | 0.700 | 1.11 (0.91-1.35) | 0.298 |
| miR500a | 1.01 (0.96-1.07) | 0.575 | 1.00 (0.83-1.21) | 0.979 |
| miR155 | 1.00 (0.97-1.04) | 0.884 | 1.04 (0.97-1.11) | 0.252 |
| miR146 | 1.00 (1.00-1.00) | 0.983 | 1.00 (1.00-1.00) | 0.992 |
| miR192 | 0.96 (0.86-1.07) | 0.459 | 0.87 (0.67-1.14) | 0.314 |

^§^ Adjusting for age (continuous), HBsAg (continuous), ALT (continuous), HBeAg status (positive vs negative), and interferon treatment (yes vs no).

**Supplementary Table 6.** Feature changes

| **Variables** | **HBsAg consistence**  **(n = 58)** | ***p* ^¶^** | **HBsAg loss**  **(n = 29)** | ***p* ^¶^** | ***p* between groups^§^** |
| --- | --- | --- | --- | --- | --- |
| HBsAg | -4.8 (-23.7 - 4.0) | **0.014** | -13.1 (-166.5 - -1.9) | **<0.001** | 0.018 |
| HBV DNA | -1 (-3.3 - 0.0) | **<0.001** | -1 (-1.4 - -0.5) | **<0.001** | 0.961 |
| ALT | -9.1 (-33.6 - 10.6) | **0.105** | -6.6 (-22.1 - 7.8) | 0.165 | 0.981 |
| AST | 0.1 (-14.4 - 12.8) | 0.696 | -0.2 (-8.0 - 11.0) | 0.903 | 0.754 |
| TBIL | -2.9 (-7.4 - 0.8) | **0.002** | -3.835 (-8.115 - -0.545) | **<0.001** | 0.396 |
| DBIL | -2.2 (-3.7 - -0.6) | **<0.001** | -2.875 (-3.605 - -2.15) | **<0.001** | 0.166 |
| IBIL | -0.7 (-4.0 - 1.9) | 0.229 | -0.61 (-3.98 - 1.455) | 0.168 | 0.802 |
| ALB | -1.2 (-4.3 - 0.7) | **<0.001** | -2.6 (-6 - -0.5) | **<0.001** | 0.130 |
| GGT | -3.3 (-17.2 - 1.2) | **0.009** | -7.1 (-14.25 - -0.1) | 0.010 | 0.563 |
| LSM | -0.2 (-1.5 - 1.0) | 0.747 | -1.05 (-2.3 - 0.1) | **0.015** | 0.073 |
| CAP | 9.0 (-29.0 - 42.0) | 0.255 | 21.5 (-18 - 46) | 0.171 | 0.588 |
| AFP | 0.7 (-0.3 - 1.5) | **0.032** | 0.9 (0.3 - 1.9) | **<0.001** | 0.141 |
| IL-6 | 0.1 (-1.7 - 1.0) | 0.589 | -0.9 (-1.3 - 0.1) | 0.053 | 0.299 |
| IL-8 | -1.1 (-30.9 - 11.5) | 0.383 | 0.1 (-55.6 - 49.3) | 0.739 | 0.459 |
| IL-21 | -7.9 (-24.9 - 4.8) | **0.003** | -1.6 (-18.6 - 3.5) | 0.225 | 0.372 |
| IL-23 | 17.9 (-21.6 - 63.4) | **0.028** | 31.5 (-6.2 - 79.4) | **0.002** | 0.328 |
| IL-33 | -3.7 (-6.7 - 0.0) | **<0.001** | -0.6 (-3.9 - 1.0) | 0.169 | **0.023** |
| MMP-1 | -0.4 (-1.1 - 0.8) | 0.227 | 0.2 (-0.3 - 1.4) | 0.372 | 0.083 |
| MMP-2 | 1.1 (-2.1 - 5.0) | 0.146 | -1.7 (-3.9 - 4.3) | 0.690 | 0.239 |
| MMP-3 | -0.6 (-7.1 - 3.8) | 0.401 | 0.7 (-4.0 - 4.4) | 0.756 | 0.459 |
| CXCL9 | 0.8 (-14.8 - 54.0) | 0.218 | 4.8 (-13.6 - 25.1) | 0.348 | 0.805 |
| CXCL10 | -0.7 (-15.7 - 12.3) | 0.729 | 1.0 (-13.2 - 8.4) | 0.756 | 0.791 |
| CXCL11 | -0.5 (-16.9 - 13.2) | 0.595 | 6.3 (-8.9 - 19.8) | 0.303 | 0.279 |
| CXCL13 | 0.7 (-8.1 - 16.2) | 0.349 | 0.6 (-9.9 - 12.9) | 0.825 | 0.805 |
| TNF-α | 2.1 (-1.2 - 4.4) | **0.053** | 2.8 (0.2 - 4.9) | **0.004** | 0.563 |
| TNF-R1 | 4.4 (-33.9 - 31.2) | 0.930 | 8.7 (-27.3 - 53.2) | 0.579 | 0.605 |
| IFN-γ | -2.3 (-11.0 - 2.2) | **0.016** | 0.7 (-7.5 - 9.8) | 0.534 | 0.058 |
| BAFF | 1.8 (-18.4 - 29.3) | 0.293 | 12.4 (-23.5 - 54.5) | 0.325 | 0.489 |
| CCL2 | -0.7 (-5.5 - 4.7) | 0.981 | 4.8 (-6.4 - 13.6) | 0.262 | 0.169 |
| miR375 | -0.3 (-1.9 - 0.3) | 0.356 | 0.4 (-0.9 - 2.0) | 0.467 | 0.262 |
| miR92a | 0.2 (-2.0 - 2.2) | 0.747 | 1.1 (-0.8 - 4.7) | 0.114 | 0.310 |
| miR500a | 0.9 (-0.1 - 4.5) | **0.012** | 1.0 (-1.6 - 4.6) | 0.071 | 0.935 |
| miR155 | 1.3(-0.1 - 19.2) | **0.014** | 3.8 (-0.1 - 21.7) | **0.002** | 0.672 |
| miR146 | 0.1 (-4.1 - 1.2) | 0.747 | 0.3 (-1.5 - 2.9) | 0.227 | 0.309 |
| miR192 | 0.1 (-2.9 - 2.6) | 0.787 | 1.3 (-0.9 - 3.8) | 0.237 | 0.361 |

^¶^ compared between baseline and afterward using single sample Wilcoxon’s Signed Rank test.

^§^ compared between two groups using Wilcoxon’s tests.
